# Social Box: A New Housing System Increases Social Interactions among Stallions

**DOI:** 10.3390/ani13081408

**Published:** 2023-04-20

**Authors:** Anja Zollinger, Christa Wyss, Déborah Bardou, Iris Bachmann

**Affiliations:** 1 Agroscope, Swiss National Stud Farm SNSF, Les Longs-Prés, CH-1580 Avenches, Switzerland; christa.wyss@agroscope.admin.ch (C.W.); bardoudeborah@gmail.com (D.B.); iris.bachmann@agroscope.admin.ch (I.B.); 2 Chaire bien-être animal, VetAgro Sup, 1 Avenue Bourgelat, F-69280 Marcy-l’Étoile, France

**Keywords:** stallion, individual housing, physical social interactions, social box

## Abstract

**Simple Summary:**

Aiming to improve the housing conditions of stallions, we tested the “social box” (SB), which allows closer physical contact between stallions housed individually in internal stables. The partition of the SB comprised vertical bars that allowed the horses to pass their head, neck and legs into the adjacent box stall. Eight pairs of stallions were filmed over a 24 h period in the SB and in their usual box stables, “conventional boxes” (CB), which strongly restrict tactile contact. We investigated the effect of housing in the SB on horses’ behaviour and the number and characteristics of injuries. The total duration of active social interactions was significantly higher in the SB than in the CB (51 min vs. 5 min). Positive interactions accounted for about 71% of the total duration of interactions in SB and CB. The stallions interacted more often in the SB than in the CB (113.5 vs. 23.8 social interaction sequences over 24 h). No grievous injuries were recorded. The social box appears to be a suitable and innovative solution to give singly housed adult stallions the possibility of having closer physical interactions in a safe way, limiting the risk of injuries they could inflict or be exposed to.

**Abstract:**

In domestic conditions, adult stallions are mostly housed individually in internal stables to reduce the risk of injuries during social interactions. Social deprivation in horses results in physiological stress and behavioural problems. The aim of this study was to test the “social box” (SB), which allows closer physical contact between neighbouring horses. Eight pairs of stallions (n = 16) were filmed over a 24 h period in the SB and in their usual box stables, “conventional boxes” (CB), which strongly restrict tactile contact. The effect of housing in the SB on behaviour and the occurrence and characteristics of injuries was investigated. The total duration of active social interactions was significantly higher in the SB than in the CB (51.1 vs. 4.9 min, *p* < 0.0001). Positive interactions accounted for about 71% of the total duration of interactions in SB and CB stabling. The stallions interacted significantly more often in the SB than in the CB (113.5 vs. 23.8 social interaction sequences over 24 h, *p* < 0.0001). No grievous injuries were recorded. The social box appears to be a suitable solution to give adult stallions the possibility of having physical interactions. Therefore, it can be considered a substantial environmental enrichment for singly housed horses.

## 1. Introduction

Horses are highly social animals. In their natural environment, feral horses (*Equus caballus*) and Przewalski horses (*Equus przewalskii*) mostly form harem bands and male bands. Harem bands comprise one or, more rarely, several adult stallions with one to several adult females and their yearlings and foals [1,2,3]. When they reach sexual maturity (at the age of 1–2 years for the females and 2–3 years for the males), the youngsters are chased out of the harem or go away on their own initiative [3]. Young mares usually join an existing harem or bind with a stallion to create a new family band. Young males and older males that have lost their own harem form so-called bachelor bands. These comprise 2 to 15 or more males and are less stable than family bands [4]. Life in a bachelor band, especially agonistic and ritualised interactions as well as play behaviours, allows males to develop the social skills and physical fitness needed to obtain a harem and defend it against rival conspecifics and predators [4,5,6]. It is widely accepted that well-developed equine body language and ritualised behaviour patterns enable stallions to exchange information about their social rank and minimise the risk of injuries during physical encounters [2,7].

Animal welfare is not only about reducing negative experiences or states of animals, but also about including elements which promote positive experiences [8,9,10]. The possibility to express appropriate normal behaviour and to have pleasurable experiences are two of the “five provisions” (updated from the “five freedoms” paradigm) for promoting animal welfare. Providing sufficient space, proper facilities, company of conspecifics, and promoting pleasure, interest, a sense of control, or engagement in rewarding activities are some examples of modern animal welfare aims [11].

In addition to free movement and continuous high-fibre feed intake, the possibility of interacting and maintaining affiliative relationships with conspecifics is vital to the horses’ wellbeing and can be considered an ethological need [12]. Studies have shown that group housing has many advantages on the development of young or recently weaned horses’ behaviour, such as reducing aggression, responding better to initial training and showing a lower frequency of unwanted behaviours (see [13] for a review). Adult horses housed in groups showed a lower level of emotionality in a novel object test [14], were easier to handle [15] and expressed less stress-related behaviours [16]. Spatial and social restrictions are usually confounded in single housing. Only a few studies have investigated the impact of these two factors independently. For example, a study conducted in France [17] aimed to identify housing factors that could alleviate the detrimental effects of single housing on horse’s welfare. The results showed that the presence of narrow metal bars allowing restricted tactile contact between neighbouring horses was not sufficient to positively influence the expression of four behavioural indicators of a compromised welfare state (stereotypies, aggressive behaviours, withdrawn posture and alert posture).

The number of horse owners willing to house their animals under more species-appropriate conditions is increasing and housing systems that allow individually housed horses to have physical interactions with conspecifics are becoming more common [18]. This can be achieved, for example, with individually housed horses being turned out in a group during the day, with a stall partition without metal bars on the upper part or with individual paddocks with non-electrified fences allowing closer contact between the horses. Over the last decade, the proportion of group-housed geldings and mares has increased gradually in Western Europe, for example, in Switzerland [18] and in the Nordic countries [19].

Housing young and mature stallions in a group under suitable conditions (stable group composition, large pasture, away from mares and unshod) has been demonstrated to be possible in a domestic context without causing grievous injuries [20,21,22,23,24]. However, adult stallions are mostly housed in single stables and turned out in individual paddocks with extremely limited physical contact or no tactile interactions with conspecifics at all. The main reasons for isolating horses are as follows: prevention of injuries linked to ritual and aggressive social interactions (especially for stallions), lack of space, high turnover with frequent departure/arrival of new horses, sanitary reasons and the need to have the horse available at all times for breeding and/or training. Some of these reasons are, indeed, only partially compatible with group housing.

Isolation and lack of social interaction in adult males, associated with other concomitant factors, such as limited access to pasture and reduced amount of forage, could explain why stereotypies are more frequently displayed by stallions than by geldings and mares (see [25] for a review of behavioural problems in stallions). Self-mutilation, such as flank biting, has been reported to occur mostly in stallions. The incidence of this behaviour was reduced when the stallions were provided with more species-appropriate housing conditions, in particular the presence of a stall companion [26]. As highlighted by two recent reviews on stallion housing [24,25], providing stallions with the possibility to have direct social interactions and maintain social bonds with conspecifics is a key point in supporting their wellbeing and preventing behavioural problems in adult stallions.

The level of aggression in horses living in groups has been shown to diminish with the increase in enclosure size [27]. To minimize the risk of injuries related to social interactions, group housing requires more space than individual stabling for the same number of horses. Due to the limited space available in some traditional stud farms, not all stallions can be housed in groups. Therefore, it is necessary to develop and assess new solutions to improve the housing conditions of individually housed stallions.

The aim of this study was to test a new partition that allows closer physical contact between stallions housed individually in internal stables (usually referred as “box stables” or “box stalls” in Continental Europe). The investigation focused on the impact of this “social box” (SB) on social interactions, activity budget and injuries. The consequences on the stallions’ social behaviour when driven in front of the carriage in pairs were investigated in a further study [28]. Eight pairs of stallions were filmed over a 24 h period in the social box and in their usual box stables, the “conventional boxes” (CB). The following questions were addressed: (a) How often and for how long do stallions express social interactions in the SB stabling compared to the CB stabling? (b) Is there a difference in the valence of the interactions in the SB stabling and in the CB stabling? (c) Does the stabling influence the activity budget of the stallions? (d) What kind of injuries can be recorded in both SB and CB stabling? Where are they located and how severe are they?

## 2. Materials and Methods

This study was carried out at Agroscope, Swiss National Stud Farm in Avenches, Switzerland, in full compliance with national rules and regulations under permit number VD 2810. The first group of 8 stallions was observed from February to April, the second group of 8 stallions was observed from October to December 2014.

### 2.1. Animals and Husbandry

Sixteen Franches-Montagnes stallions aged 3 to 14 years (mean age ± SD = 9.3 ± 3.2) and 154 to 160 cm (157.7 ± 1.7) in height, owned by the Swiss National Stud Farm (SNSF), were included in this study. As prescribed by Swiss animal welfare legislation, they were all reared in groups with conspecifics from the time of weaning to at least the age of 30 months. Since their arrival at the SNSF at the age of 3 years, all stallions had been housed individually in a CB (detailed description below). They were fed hay and concentrates 3 times daily and kept on long straw bedding except for two horses, who were kept on sawdust bedding because of respiratory problems. Water was available ad libitum by automatic drinkers. All stallions were turned out in individual paddocks (without tactile contact to conspecifics) for about 2 h every day and were trained (ridden, driven and exercised in a horse walker) 4–5 times a week. They were all shod, clinically healthy and, according to Swiss animal welfare legislation, their vibrissae were not trimmed. All stallions included in this study were approved breeding stallions. However, none of them was mating at the time of data collection. The last mating occurred at least 7 months (first group observed) or, respectively, 2 months (second group observed) before the start of the study. Prior to data collection, the stallions might have been housed on several occasions in adjacent box stalls and/or hitched next to each other when driven in front of the carriage. Thus, they might have “known” each other but never had the possibility of unrestricted physical contact.

### 2.2. Experimental Design

**Figure 1:**
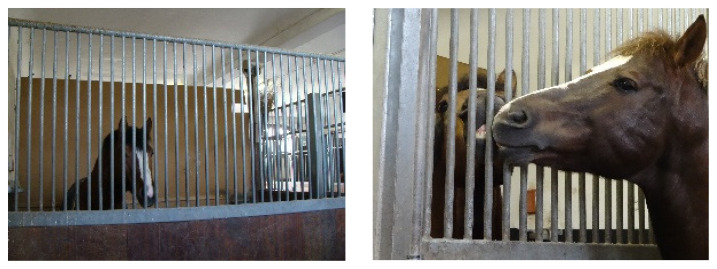
“Conventional box” stabling (CB) allows only restricted tactile contact.

**Figure 2:**
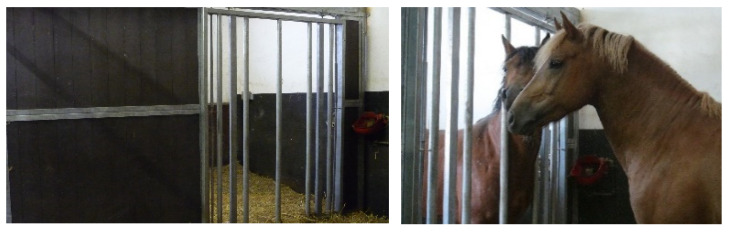
“Social box” stabling (SB) allows closer physical contact.

The partition of the CB comprised a lower solid wooden part (1.40 m high) and an upper part (another 1.15 m high) with vertical metal bars spaced at 5 cm, allowing visual, auditory and olfactory contact but strongly restricting tactile contact (Figure 1). The partition of the SB was originally designed by Andreas Kurtz (“Kurtzbox”, Animal consulting Switzerland, https://animalconsulting.ch, (accessed on 24 February 2023)). It comprised one part with vertical metal bars (from the ground to a height of 2.55 m) spaced at 30 cm, allowing the horses to pass their head, neck and legs to the adjacent box stall. The second part of the partition was solid, allowing the horses to visually isolate themselves from the neighbouring horse if they wanted to (Figure 2). The floor area of every internal stable was 9.3 m^2^.

**Figure 3:**
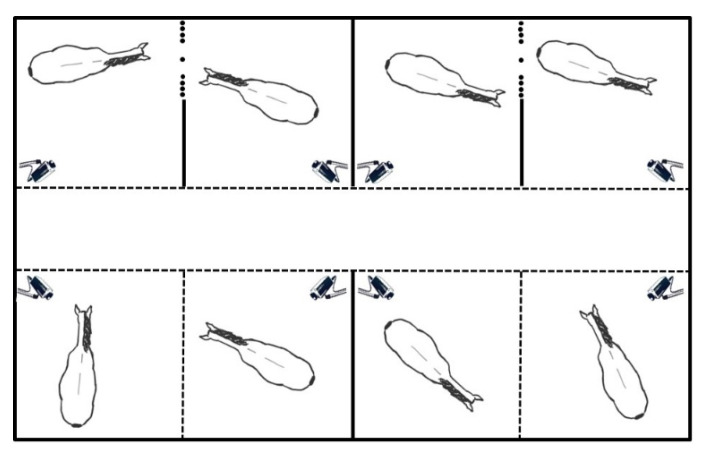
Experimental stables with 2 × 2 social boxes (above) and 2 × 2 conventional boxes (below). An opaque partition prevents visual and tactile contact between the two sets of box stalls, thus allowing tactile contact only within the same pairs.

The experimental stable was arranged in one row of four SBs facing another row of four CBs. Each set of two consecutive box stalls was separated from the next set of box stalls by an opaque partition (Figure 3) so that each stallion could physically interact with only one neighbouring stallion. Openings situated over the box stall’s doors enabled every stallion to put their head into the corridor and to see his conspecifics stabled on the opposite side. The sixteen stallions were randomly divided into two groups of eight horses and randomly assigned to a CB or an SB in the experimental stable. Thereafter, these pairs of neighbouring stallions remained unchanged throughout the study. Each stallion was stabled for 20 days in a CB preceded or followed by 20 days in an SB.

### 2.3. Behavioural Data Collection

Each stallion was filmed over a 24 h period on days 19 and 20 in each CB and SB, using a surveillance camera (Panasonic WV SW316 LE, HD resolution of 1280 × 960 at 30 fps, integrated infrared spotlight) mounted high on the walls in each box stall in order to gain an adequate visual field. A total of 768 h of videos were recorded. The behaviour of each stallion in both CB and SB stabling was encoded from the videos by a trained ethologist using The Observer XT v.11.5 software (Noldus Information Technology, Wageningen, The Netherlands). Videos were analysed in a randomised order.

#### 2.3.1. Social Interactions

The social behaviour of two neighbouring stallions was encoded simultaneously (two videos were played synchronised side by side) using the continuous sampling method. Each sequence of a social interaction was recorded as a state event. A social interaction sequence was defined as starting when a stallion expressed a social behaviour listed in the ethogram (Table 1) towards his neighbour and ended when both stallions changed their activity towards another behaviour, such as feeding. Thus, the number of social interactions and the duration of each social interaction sequence were calculated for each pair of stallions. Each social interaction sequence comprised one or several distinct behaviours, which were coded as point behaviour and reported as frequency. The ethogram had been compiled from observations of other stallions housed in the experimental stable during a previous large pilot study and with literature research. Only active and clearly identifiable social interactions, often involving tactile contact, were included in the ethogram. Although spatial proximity with a conspecific has been shown to be an important factor when studying social relationships [29,30,31], behaviours such as “resting side by side” or “eating close together” were not included in the current study, as the aim was to focus on the potential impact of the new partition on physical, potentially dangerous interactions between singly housed stallions.

Finally, each social interaction sequence was assigned a positive, negative or unknown valence. A social interaction was determined to be positive if the initiator’s ears were mainly in the forward position or if the receiver responded to an approach by moving towards the initiator, engaging in reciprocal play [32], and the observed behaviours within a sequence were of predominantly affiliative nature such as sniff, nibble, play, which may indicate a desire for friendly interaction and proximity [30,32]. If an approach elicited a retreat by the approached animal or mainly agonistic and aggressive behaviours, such as threatening to bite or actual bite or threatening to kick [4,30] with ears often laid back, the interaction was assigned a negative valence. If the social behaviours within a sequence were difficult to attribute to a predominantly positive or negative valence, this sequence was assessed to be of unknown valence.

#### 2.3.2. Activity Budget

All the videos were analysed a second time to create an activity budget for both SB and CB stabling using the scan sampling method, with a recording interval of 5 min over 24 h per horse. The following activities were recorded: feeding (including exploratory behaviours related to feed intake), standing (including resting), sternal recumbency, lateral recumbency, moving (excluding single steps during feeding), maintenance (e.g., drink, defecate, masturbate, roll, self-grooming) and social interaction. Data were collected only when the horse was in the stable, free to move, without being handled by a human.

### 2.4. Injuries Data Collection

Pre-existing injuries were recorded as soon as the stallions were housed in the experimental stable (day 0) as a baseline. Each additional injury was recorded by a veterinarian on the same data collection sheet on days 2, 4, 11 and 18 of the stay in a CB and an SB. All injuries were recorded, including those that were obviously not attributable to social interactions (e.g., related to itching or caused by poorly fitted riding or carriage driving equipment).

**Figure 4:**
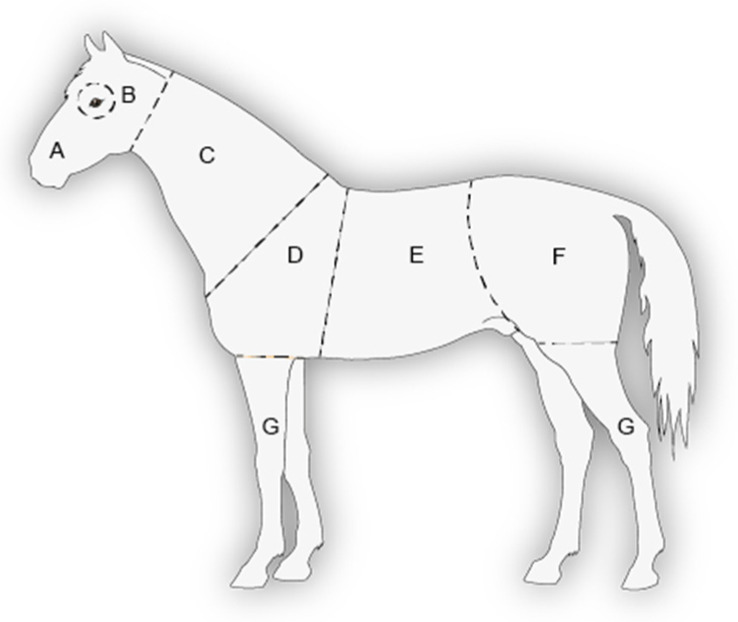
Horse body sketch used to record the location of the injuries, adapted from [34,36,37]. The letters correspond to the following body areas: A—Head; B—Eyes; C—Neck; D—Shoulders; E—Barrel; F—Rump; G—Legs.

An injury scale was developed to categorise the severity of every injury (Table 2). The injury’s location on the horse’s body was noted on a sketch with defined body areas (Figure 4). The size (categorised as follows: S: <2.5 × 2.5 cm; M: <5 × 5 cm; L: <10 × 10 cm; XL: <15 × 15 cm) and an indication of whether treatment of the wound was required were also noted.

**Table 2:** Injury scale used to categorise the severity of the injuries, adapted from [33,34,35,36]. The presence or absence of blood or scab was the main factor used to categorise injuries.

Category	Description
1	Minor blemishes involving scratched and/or missing hair with no observable skin lesion, no change in the skin pigmentation, slightly swollen area, not painful to the touch.
2	Blemishes involving missing hair with skin scuff on the epidermis (skin irritation, redness or loss of skin pigmentation). No traces of blood, no scab.
3	Superficial skin lesion on the dermis, skin nick, traces of blood and/or thin scab.
4	Open wound involving subcutis, visible deeper tissue (e.g., muscles, tendons), bloody or presence of a relatively thick scab. No suture required.
5	Deep open wound, visible tendon and/or bone structure, severe cut through the skin that requires stitching because of wound depth and/or size.
6	Severe injury that may cause a long-lasting loss of function (e.g., laceration with extensive soft tissue damage, seriously injured tendon, serious joint damage, fracture).

### 2.5. Data Analysis

#### 2.5.1. Social Interactions

Statistical analyses were performed using the R software (version 4.1.1, R Core Team, 2020). The significance level was set at *p* < 0.05. Linear mixed-effects models (LMM, the lmer function from the lme4 package) were carried out to test the effect of SB vs. CB stabling on social interactions. The models included the total duration of interactions (in minutes), the mean duration per interaction (in seconds) and the occurrence (in number) as a response variable.

The fixed effect variables were the valence (positive, negative, unknown), the stabling (SB, CB), the order of data collection (SB first, CB first), the group (namely the season in which the data collection took part: February–April, October–December) and the interaction between these factors. The order of data collection and the group were not significant, and were therefore excluded from the model. The horse pair nested within the group was included as a random effect. Thus, each horse pair served as its own control when stabled in CB.

For all models, the residuals were graphically checked for normal distribution and homoscedasticity (hist, qqnorm and plot functions). Log transformation was applied to satisfy assumptions. All models were fitted with a restricted maximum likelihood (REML) estimation. The *p*-values were calculated based on Satterthwaite’s approximations (ANOVA function). Non-significant factors and interactions were removed to obtain the final models. When a significant interaction effect was found in the final model, further two-by-two comparisons were performed using Wilcoxon signed rank tests (WLX, wilcox.test function or wilcoxsign_test function; the coin package in case of ties) or Tukey tests (TKY, glht function, multicomp package).

#### 2.5.2. Activity Budget

The percentage of scans for every activity was calculated by combining sternal and lateral recumbency to limit multiple testing. The effect of SB vs. CB stabling on the activity budget was then analysed using a paired WLX.

#### 2.5.3. Injuries

The effect of stabling on the number of injuries was analysed using a paired WLX. Due to the small sample size, data related to the injuries’ severity, location and size are presented descriptively.

## 3. Results

### 3.1. Behavioural Data

#### 3.1.1. Social Interactions

##### Total Duration

Over a 24 h period, the total duration of social interactions, all valences combined, averaged 51.1 ± 24.4 min in SB and 4.9 ± 2.9 min in CB (Table 3). There was an effect of the stabling on the total duration of social interactions (LMM, X^2^ = 70.10, Df = 6, *p* < 0.0001). Post hoc comparisons showed that the total duration of social interactions was significantly higher in SB compared to CB for positive valence (WLX, Z = 2.52, *p* = 0.012), negative valence (WLX, V = 36, *p* = 0.008) and unknown valence (WLX, Z = 2.52, *p* = 0.012). The proportion of the total duration of social interactions by valence was similar in both SB and CB stabling. Positive interactions accounted for about 71% of the total duration of interactions.

##### Occurrence and Duration per Interaction

Over 24 h, the occurrence of social interactions averaged 113.5 in SB and 23.8 in CB. There was an effect of stabling on the total number of social interaction sequences (LMM, X^2^ = 58.16, Df = 6, *p* < 0.0001). Post hoc comparisons showed that the occurrence of social interaction sequences was significantly higher in SB compared to CB for positive valence (WLX, Z = 2.52, *p* = 0.012), negative valence (WLX, V = 36, *p* = 0.014) and unknown valence (WLX, V = 36, *p* = 0.008).

All valences combined, the mean duration per interaction was 27.0 ± 55.8 s in SB and 12.4 ± 13.8 s in CB (Table 4). There was an effect of stabling on the mean duration per interaction (LMM, X^2^ = 29.02, Df = 6, *p* < 0.0001). Post hoc comparisons showed that social interaction sequences were significantly longer in SB than in CB for positive valence (WLX, V = 36, *p* = 0.008) and unknown valence (WLX, Z = 2.5205, *p* = 0.012). However, there was no significant difference in the duration per interaction for negative valence (WLX, Z = 1.26, *p* = 0.207) in SB compared to CB.

The distinct social behaviours listed in the ethogram were coded as point behaviour within each social interaction sequence. In the SB, the stallions expressed 23 different social behaviours compared to 18 in the CB. Thus, the stallion’s behavioural repertoire increased by almost 27.8% when housed in SB.

In the SB, the most frequently observed behaviours (i.e., more than 5% of the occurrences) during direct mutual social interactions were evasive balk (22.8% of occurrences of point behaviour), nip (21.3%), sniff (11.2%), nuzzle (10.3%), repel (7.8%), ignore (7.3%) and approach (4.8%). In the CB, the most frequently observed social behaviours were sniff (37.5% of occurrences), ignore (13.0%), repel (11.2%), approach (10.1%), attempt to nuzzle (10.1%) and attempt to nip (5.0%).

#### 3.1.2. Activity Budget

A total of 256.8 ± 19.1 (mean ± SD) scans per horse were recorded in SB stabling and 256.5 ± 19.0 per horse in CB stabling. Variations resulted from the duration of the horse being outside the stable (for turnout and training) or not being free to move due to the presence of a human in the stable. Thus, the recorded scans covered an average of 21.4 h per day for both SB and CB stabling. The only significant difference in activity budget between SB and CB was for social interactions (WLX, V = 0, *p* < 0.0001). On average, stallions expressed social interactions in 3.8% of the recorded scans in SB and in 0.4% in CB (Table 5). The distribution of other activities was similar in both SB and CB stabling (WLX, *p* > 0.08). This result suggests that the social interactions recorded in SB stabling were not systematically expressed at the expense of another specific activity.

### 3.2. Injuries

The 16 stallions were examined four times over a period of 20 days in both SB and CB stabling. A total of 140 injuries were recorded. The total number of injuries was higher in SB (115 injuries) than in CB (25 injuries) (WLX, Z = −3.262, *p* < 0.0001). In the SB, stallions had 1–19 injuries (7.2 ± 4.6). In the CB, stallions had 0–7 injuries (1.6 ± 1.9). Of the total, 82% (n = 95) of the injuries observed in SB and 84% (n = 21) of the injuries observed in CB were scratched and/or missing hair with no observable skin lesion (Category 1) and hair loss, skin scuff, skin irritation, no traces of blood and no scab (Category 2). Injuries involving skin lesions, traces of blood or/and thin scab (Category 3) were observed in a similar proportion in both SB and CB stabling: 17% (n = 20) in SB and 16% (n = 4) in CB. No injury was assigned to Categories 4, 5 and 6 (Table 6).

In SB, the largest proportions of injuries were those recorded on the head (40%) and around the zygomatic arch above the eyes (33%). These two body areas accounted for the largest proportions of injuries in Categories 2 and 3 (Table 7). In CB, injuries were also mainly located on the head (32%), followed by the legs (28%) (Table 8). No injuries were recorded on the neck in CB compared to 9 injuries in SB.

Out of the entire data set, four injuries required treatment. Three of these, recorded in the SB, were located near the eye, and were therefore treated with anti-inflammatory eye drops. The fourth injury was a self-injury of the bulb recorded in the CB and treated with disinfectant healing cream. Of all the injuries, 95% were in the size category S (<2.5 × 2.5 cm). Four injuries, two recorded in the SB and two recorded in the CB, were in the category M (<5 × 5 cm). None of these injuries required medical treatment.

In the 768 h of video recordings, one stallion was observed to have been cast against the concrete wall for 233 s, and two stallions were caught in the metal bars for 38 and 49 s, respectively. These situations occurred when the horses rolled in the straw and ended up with the legs folded up against the wall or between the metal bars. The animals managed to get up without the help of a human.

## 4. Discussion

### 4.1. Behavioural Data

In the SB, with all valences combined, the total duration of social interactions increased by a factor of 10 to an average duration of 51 min over 24 h compared to 5 min in the CB. In the present study, only active social interactions, often involving tactile contact, were recorded. Passive interactions such as spatial proximity—which often have a long duration—were not considered. Regarding the activity budget, social interactions represented 3.8% of the recorded scans in the SB compared to 0.4% in the CB. With an average daily occurrence of 113 social interaction sequences compared to 24 occurrences in the CB, the SB encouraged the stallions to initiate a social interaction sequence more often. The possibility of socially interacting can be considered a substantial environmental enrichment [38] for individually housed stallions.

The total duration of social interactions with a positive valence represented more than 70% of the interactions in both SB and CB stabling. A similar percentage was observed in the Przewalski bachelor band [2]: the proportion of non-agonistic social interactions was 63%, and more than half of these interactions were considered friendly. The mean duration of the social interaction sequences with a positive valence was higher in the SB than in the CB. This can be explained by the fact that play sequences, which were assigned a positive valence, usually involve a chain of behaviours, such as sniff, followed by nuzzle and nip interspersed with repel and avoidance. Tactile behaviours, such as nuzzling and nipping, cannot be expressed in the CB because of the narrow space between the bars (5 cm) of this type of partition. Thus, the behaviours remained at an attempt stage and the sequences of play behaviours were interrupted. If an animal is motivated to express a behavioural chain but is unable to do so, it could result in displacement or redirected activities. If the problem is repeated or chronic it could lead to stereotypical behaviours, such as crib biting or wind sucking (see [39] for a review on stereotypies).

Play is often considered an important factor in the behavioural, social and physical development of an individual and is thought to strengthen the bond between individuals [32,40]. Wild und feral immature male horses are playful, but play is practically absent in the social behaviour of adult stallions [5]. Some authors have suggested that play in mature animals, using the horse as an example, may indicate frustration resulting from disturbed welfare, and does not necessarily reflect a positive emotional state [41,42]. In the present study, play sequences were assigned a positive valence because they are composed of several distinct affiliative behaviours that are not intended to injure the conspecific. Play in adult stallions could also be considered an important possibility to redirect frustration to a behaviour with rewarding character. It may represent a way for adults to relieve stress [41].

The total duration of social interactions with a negative valence represented 13–14% of the interactions in both SB and CB stabling. Under natural conditions, the frequency of assessed agonistic behaviours in bachelor bands is highly variable, depending on the populations studied and the related parameters (number of horses, pen size, data collection method, etc.). Several authors have reported that the rate of aggression is low and agonistic interactions rarely result in actual fights that might cause serious injury [2,6,43]. Horses, like many other species, favour agonistic behaviours of the minimum intensity required by the situation [44]. Low-intensity aggressive behaviours, such as bite threat or kick threat, are preferred over high-intensity aggressive behaviours, such as bite or kick [36]. An increase in the number and intensity of aggressive interactions in stallions is generally induced by limited resources (e.g., additional food [2] or shade [43]) and by the presence of mares.

Several authors have reported that the proximity of mares increases the occurrence of aggressive behaviours in harem stallions towards stallions from other harems or from other bachelor bands [2,6,45]. Experts advise to house stallions out of view of the mares to reduce stress, especially during the breeding season [46]. In the present study, there were no mares within 300 metres, and the stallions were not mating at the time of data collection. This could explain why the statistical analysis did not reveal a difference related to the season (i.e., observations carried out on the first group, from February to April, during the breeding season vs. observations carried out on the second group from October to December, outside the breeding season). This could further explain the overall low proportion of negative social interactions. The feasibility of housing stallions in SB in the presence of mares in the same stable or when the stallions have a simultaneous breeding activity could be the subject of future study.

Space restriction and lack of socialisation at a young age are additional factors that increase aggressive interactions [20,27]. Even if they had been housed in CB since 3 years of age, all stallions included in the present study were reared in groups, and thus were able to develop social skills with conspecifics. Due to the very small floor area provided for each horse in internal stables, it seemed important that one part of the partition of the SB be solid to allow the horses to visually isolate from the neighbouring horse, as they were unable to spatially move away from each other.

There was no difference in the mean duration of the social interaction sequences, with a negative valence in the SB compared to the CB. Negative social interaction sequences were short. They lasted less than 10 s in both SB and CB stabling and comprised behaviours aimed at stopping the interaction. In contrast, during interaction sequences, such as play fighting (considered a positive valence), the horses alternate offensive and defensive roles and thus prolong their interaction [32].

During their stay in the SB stabling, the repertoire of social interactions increased by almost 24% compared to the social interactions during their stay in the CB stabling. Therefore, this new housing system allows horses to express a wider range of normal social behaviours. Findings from a study conducted with young stallions divided into two treatment groups: singly stabled and group stabled suggests that full-scale physical contact is necessary for the formation of associative relationships between horses [20]. In the cited study, as both treatment groups were joined and turned out in a common pasture, the previously group-stabled stallions were found to seek proximity with their previous group members. In contrast, the previously individually stabled stallions did not associate more with their previous box stall neighbour [20]. Future studies should investigate whether SB stabling, allowing stallions to express a wider range of tactile interactions but still not the full scale of the social ethogram, could help horses to form associative relationships between conspecifics.

When living in a group, horses usually have one or more preferred partners for affiliative relationships, such as mutual grooming and spatial proximity (see [29] for a review). In this study, the neighbouring stallions were paired randomly. In practical reality, it is recommended to consider an affiliative relationship between individuals when housing horses in adjacent box stalls. More research is necessary to better predict affiliative relationships between individuals and minimise the risk of potentially dangerous aggressive interactions in housing systems with opportunities for physical contact.

The vertical metal bars of the SB provide a possibility for singly housed stallions to express a wider range of social behaviours and to engage in behaviours they may find rewarding, for example affiliative interactions, such as play behaviour [8,47]. The second part of the SB’s partition being solid allows the stallions to choose to visually isolate themselves from the neighbouring horse. The possibility to express a wider range of natural behaviour, have positive experiences, and gain more control over their own environment can contribute to the welfare of the horses.

### 4.2. Injuries

No grievous injuries were found over the six weeks of data collection. In both SB and CB, over 80% of the injuries were scratches, hair loss, skin scuff and skin irritation with no trace of blood, thus carrying a low risk of infection. Injuries involving a lesion of the skin were mainly situated around the eyes in the SB and on the head in the CB. These findings are examined in greater depth below.

As group housing for stallions is unusual in the domestic context, injuries related to social interactions are poorly documented. In two studies conducted on domestic stallions housed in groups, only a few minor injuries were recorded, despite intensive social interaction when first grouped [20,21]. Under natural conditions, almost all feral adult stallions observed in the Great Basin in western North America had bite-related wounds [6,45]. Many authors state that social interactions between stallions are largely ritualised and that serious injuries caused by aggressive interactions within bachelor bands in both feral and wild horses are rare, even in real combat [6,43]. However, fights between stallions can also cause life-threatening injuries. For example, a feral Konik Polski stallion had his lip bitten from a conspecific and could no longer feed properly [48].

In the SB, the vast majority of injuries were located on the head and around the eyes. This result is not consistent with previous findings, where injuries were mainly found on the barrel and the rump in semi-feral groups with males and females of all ages [34] and in group-housed riding school horses with females and geldings [36]. This difference could be attributable to sex, with stallions having more intense social interactions and of a different nature than mares and geldings. However, video recordings and direct observations revealed that the injuries located above the eyes around the zygomatic arch in the SB were not directly caused by the neighbouring horse. They occurred when the stallions bumped their heads against the metal bars during social interactions, especially during sequences with behaviours such as rear, bite (and bite threat), nip, head shake, evasive balk and avoidance, regardless of the valence of the social interaction sequence.

Injuries caused by hitting the metal bars should be avoided by using a suitable material to pad the bars. The results of additional tests carried out at the Swiss National Stud Farm with hard plastic tubes over the metal bars appeared to be promising to avoid injuries around the eyes. Additional information can be requested from the authors.

Injuries recorded in the CB were obviously not caused by social interactions but rather by itching or poorly fitted riding or carriage driving equipment (injuries related to the noseband, the girth or the leg boots). Even if the injuries related to social interactions are not serious, they can be aesthetically undesirable when stallions are presented in breeding events and equestrian shows or participate in equestrian competitions. There is a need to better educate stallions’ caretakers, riders, judges and the public, explaining that these minor injuries are a natural consequence of stallions having the possibility to interact socially with conspecifics.

When designing a partition that allows closer physical contact between stallions, special attention should be paid to the height of the openings and to the material used to pad the metal bars to minimise injuries located on the head and around the eyes of the horses. The space between the vertical metal bars has to be adapted to the width of the horse’s chest in order to ensure that the animals can only pass their head and neck but not their shoulders to the adjacent box stall.

The size of the box stall may also be an important factor to consider. In the present study, every internal stable measured 9.3 m^2^, which is relatively small for horses measuring 154 to 160 cm in height [49,50]. The risk for the horse to be casted against the wall, or to get caught in the metal bars with their legs after rolling, could be reduced by providing more space [51]. Two years ago, a private equine stud farm in Germany successfully installed the SB with 25 m^2^ floor area per box stall for their German Warmblood breeding stallions (Alexandra Gasser, Gut Schönweide, personal communication, 2021). Further scientific studies could test the SB with other breeds. It might also be interesting to investigate whether the SB would be an appropriate transitional solution to prepare socially deprived horses for reintegration into group housing as suggested by a pilot study on stallions conducted in France [52].

### 4.3. Limitations

The present study focused on the impact of the SB on social interactions, activity budget and injuries. The study design does not allow us to state whether the stallions were in a better state of welfare when housed in the SB compared to the CB. It would have been interesting to assess the emotional state of the animals, for example by using a cognitive bias test, which has previously been used to compare different housing conditions in horses [53,54]. Physiological and behavioural welfare indicators could also have been assessed to give an indication of the altered or improved welfare state of the stallions when housed in the SB and in the CB (see [55] for a review).

The methodology applied to record injuries did not allow evaluation of their cause, such as social interactions (biting, kicking), hitting the metal bars or poorly fitted riding equipment. Future research should therefore record injuries several times a day, and every horse should systematically be checked before and after each training session, and each turnout in the paddock.

The current study tested the SB with 16 stallions of the same breed, housed and trained under similar and controlled conditions. It would be interesting to evaluate the SB with other breeds, and to investigate the effect of additional factors that might influence the social behaviour of the horses, such as feeding management, mating activity, presence of mares, training stress, pain, socialization at young age, as well as duration and frequencies of turnouts.

## 5. Conclusions

The results of this study showed that neighbouring stallions made use of the possibility of mutual physical interactions offered by the SB. They extended their behavioural repertoire and increased the duration of their social interactions tenfold compared to the CB. This possibility of socially interacting can be considered a substantial environmental enrichment and could help stallions to relieve stress induced by single housing.

The number of injuries was higher in the SB than in the CB, but no grievous incidents were recorded. The vast majority of blemishes were missing hair and skin irritation. Injuries located on the head and around the eyes of the horses could be easily reduced with padding the metal bars.

Thus, the SB appears to be a good innovative solution to give adult stallions the possibility of having closer physical interactions in a secure way, limiting the risk of injuries they could inflict or be exposed to.

## Figures and Tables

**Table 1:** Ethogram of social behaviour recorded as point behaviour, adapted from [2,4,7,32].

Behaviour	Description
Approach	With ears forward or to the side, the horse directs its body or moves toward the other horse.
Avoidance	The horse increases distance to the other horse by moving away.
Back up	The horse is moving backward. The diagonal pairs of legs move almost simultaneously.
Bite	The jaws are open widely, closed quickly with actual dental contact to the flesh or hair of the other horse’s body. The ears are laid back and the lips are retracted so that teeth are visible. In the conventional box stabling, Bite was coded when the horse actually bit in the metal bars.
Bite threat	Bite intention movement toward the other horse with ears laid back and neck extended with no actual contact. Ears are laid back, jaws are open, and lips are often retracted so that teeth are visible. Sometimes, the horse mimics a bite in the air.
Evasive balk	The horse avoids contact with the other horse by turning its head, its neck and sometimes its forehand while the hind legs remain or pivot in place.
Groan	The horse vocalises a low-pitched guttural sound.
Head shake	The horse shakes or swings its head. It can be a head bowing with alternate flexion and extension of the neck but also a lateral flexion of the neck.
Hindquarter turn	The horse turns his rump toward the other horse while forelegs remain immobile.
Hindquarter withdrawal	The horse withdraws its croup from the contact of the other horse while forelegs remain immobile.
Ignore	The horse shows no reaction to the social behaviour expressed by the other horse and continues what it was doing (for example: eating or resting).
Kick	One or both hind legs are lifted off the ground and quickly extended backward toward the other horse, with or without actual contact with the other horse’s body.
Kick threat	One or both hind legs are lifted off the ground without being extended backward toward the other horse.
Leg withdrawal	The horse holds one leg in the air, standing on three legs.
Nip	The jaw and teeth are opened and closed slightly taking a small piece of hair or flesh of another horse between the teeth. In the conventional box stabling, Nip was coded for every nip attempt through the metal bars.
Nuzzle	The horse explores a body area of the other horse with its lips. Jaws are closed, with no dental contact. In the conventional box stabling, Nuzzle was coded for every nuzzle attempt through the metal bars.
Paw	One front leg is lifted from the ground, then extended quickly in a forward direction, followed by movement backward dragging the hoof against the ground in a digging motion. The movement of the leg is horizontal rather than vertical and may be repeated several times in a row. The hoof may or may not touch the ground. Paw was coded as a default behaviour when the observer was not able to decide between Paw and Stomp.
Push	The horse is pressing one of its body parts against the other horse in an apparent attempt to make it move away. In the social box and conventional box stabling, Push was also coded when one horse pushed against the partition behind which the other horse was standing.
Rear	The hind legs remain on the ground while the forehand is raised into the air a few centimetres to a very high, nearly vertical position.
Repel	The horse walks toward the other horse with the head partly or completely toward the other horse, ears laid back. This results in the other horse moving away.
Sniff	The horse sniffs the nose, neck, flank or any other body area of the other horse, which may or may not reciprocate. Sniff was coded as the default behaviour when the visual field on the video did not allow distinguishing a movement or opening of the jaw.
Squeal	The horse vocalises a long, high-pitched laryngeal sound usually following sniffing.
Stomp	One foreleg or one hind leg is raised and lowered quickly and firmly strikes the ground. The movement of the leg is vertical rather than horizontal. Often associated with vocalisation during ritualised interactive sequences.

**Table 3:** Total duration (mean ± SD (range)) in minutes of social interactions over 24 h in the social box (SB) and the conventional box (CB) stabling by valence. The percentage indicates the proportion of the total interactions’ duration by valence.

	Total Duration [Minute]			Proportion [%]		
Valence	SB	CB	*p*-Value	SB	CB	*p*-Value
Positive	36.3 ± 17.2 (8.3–56.3)	3.8 ± 3.1 (1.0–10.9)	0.012	71.2 ± 14.9 (42.3–89.5)	71.9 ± 26.6 (34.2–97.0)	0.726
Negative	5.3 ± 2.3 (1.2–7.5)	0.5 ± 0.4 (0.1–1.4)	0.008	12.7 ± 9.6 (5.2–35.7)	14.2 ± 14.0 (1.5–40.5)	0.641
Unknown	9.0 ± 8.4 (1.3–26.2)	0.5 ± 0.6 (0.0–1.6)	0.012	16.2 ± 9.5 (5.3–31.4)	13.9 ± 16.7 (0.0–42.9)	0.742
All valences	51.1 ± 24.4 (20.2–83.7)	4.9 ± 2.9 (2.9–11.6)	<0.0001			

**Table 4:** Occurrence of social interactions (mean ± SD (range)) during 24 h and duration per interaction (mean ± SD (range)) in the social box and the conventional box stabling by valence.

	Occurrence [n]			Duration per Interaction [Second]		
Valence	SB	CB	*p*-Value	SB	CB	*p*-Value
Positive	54.3 ± 16.4 (33–103)	16.0 ± 8.5 (6–32)	0.012	39.2 ± 73.9 (1.7–765.8)	14.4 ± 15.8 (1.3–140.7)	0.008
Negative	40.1 ± 22.6 (10–82)	4.5 ± 3.7 (1–11)	0.014	8.4 ± 12.1 (1.0–166.7)	7.2 ± 5.5 (1.5–32.2)	0.207
Unknown	19.1 ± 9.5 (6–31)	3.3 ± 4.0 (0–12)	0.008	28.1 ± 32.3 (2.2–226.8)	9.9 ± 8.1 (2.6–41.7)	0.012
All valences	113.5 ± 39.9 (57–183)	23.8 ± 7.6 (15–38)	<0.0001	27.0 ± 55.8 (1.0–765.8)	12.4 ± 13.8 (1.3–140.7)	<0.0001

**Table 5:** Activity budget in the social box (SB) and the conventional box (CB) stabling presented in percentage of occurrences (mean ± SD (range)) for each activity recorded by scan sampling during the time spent in the box in absence of a human.

	Occurrence [n]		
Activity	SB	CB	*p*-Value
Feeding	51.4 ± 4.7 (40.9–62.9)	53.1 ± 6.1 (43.1–63.4)	0.433
Standing	33.6 ± 6.1 (18.8–44.3)	34.0 ± 6.3 (17.8–46.3)	0.940
Recumbency	8.7 ± 3.4 (1.8–16.2)	9.9 ± 2.6 (5.2–19.3)	0.088
Social interactions	3.8 ± 1.7 (1.1–6.9)	0.4 ± 0.3 (0.0–1.3)	<0.0001
Maintenance	1.5 ± 0.9 (0.0–3.4)	1.7 ± 0.7 (0.0–3.3)	0.562
Moving	0.9 ± 0.6 (0.0–2.4)	0.8 ± 0.4 (0.0–2.1)	0.698

**Table 6:** Distribution of injuries in the social box (SB) and the conventional box (CB) stabling by severity assessed in three categories (1 = scratched and/or missing hair, 2 = hair loss, skin irritation, 3 = skin lesions, traces of blood and/or thin scab).

	Severity of Injuries [% (n)]	
Category	SB	CB
1	32% (37)	40% (10)
2	50% (58)	44% (11)
3	17% (20)	16% (4)
4–6	0% (0)	0% (0)

**Table 7:** Distribution of injuries in social box (SB) stabling by severity and location on the horse’s body area in [% (n)].

Location—SB				
Body Area	Injuries	Category 1	Category 2	Category 3
Head	40% (46)	32% (12)	50% (29)	25% (5)
Eyes	33% (38)	16% (6)	33% (19)	65% (13)
Shoulders	11% (13)	22% (8)	7% (4)	5% (1)
Neck	10% (12)	19% (7)	7% (4)	5% (1)
Barrel	3% (4)	11% (4)	0% (0)	0% (0)
Legs	1% (1)	0% (0)	2% (1)	0% (0)
Rump	1% (1)	0% (0)	2% (1)	0% (0)

**Table 8:** Distribution of injuries in the conventional box (CB) stabling by severity and location on the horse’s body area in [% (n)].

Location—CB				
Body Area	Injuries	Category 1	Category 2	Category 3
Head	32% (8)	20% (2)	36% (4)	50% (2)
Eyes	8% (2)	0% (0)	9% (1)	25% (1)
Shoulders	4% (1)	10% (1)	0% (0)	0% (0)
Neck	0% (0)	0% (0)	0% (0)	0% (0)
Barrel	20% (7)	40% (4)	9% (1)	0% (0)
Legs	28% (7)	30% (3)	27% (3)	25% (1)
Rump	8% (2)	0% (0)	18 (2)	0% (0)

## Data Availability

Data are available upon request to the corresponding author.

## References

[1] McCort W.D. (1984). Behavior of Feral Horses and Ponies. J. Anim. Sci..

[2] Zharkikh T.L., Andersen L. (2009). Behaviour of Bachelor Males of the Przewalski Horse (Equus Ferus Przewalskii) at the Reserve Askania Nova. Zool. Gart..

[3] Feh C. (1999). Alliances and Reproductive Success in Camargue Stallions. Anim. Behav..

[4] McDonnell S.M., Haviland J.C.S. (1995). Agonistic Ethogram of the Equid Bachelor Band. Appl. Anim. Behav. Sci..

[5] Hoffmann R. (1985). On the Development of Social Behaviour in Immature Males of a Feral Horse Population (Equus Przewalskii f. Caballus). Z. Säugetierkd..

[6] Tilson R.L., Sweeny K.A., Binczik G.A., Reindl N.J. (1988). Buddies and Bullies: Social Structure of a Bachelor Group of Przewalski Horses. Appl. Anim. Behav. Sci..

[7] Christensen J.W., Zharkikh T., Ladewig J., Yasinetskaya N. (2002). Social Behaviour in Stallion Groups (Equus Przewalskii and Equus Caballus) Kept under Natural and Domestic Conditions. Appl. Anim. Behav. Sci..

[8] Mellor D.J. (2016). Updating Animal Welfare Thinking: Moving beyond the “Five Freedoms” towards “A Life Worth Living. ” Animals.

[9] Boissy A., Manteuffel G., Jensen M.B., Moe R.O., Spruijt B., Keeling L.J., Winckler C., Forkman B., Dimitrov I., Langbein J. (2007). Assessment of Positive Emotions in Animals to Improve Their Welfare. Physiol. Behav.

[10] Yeates J.W., Main D.C.J. (2008). Assessment of Positive Welfare: A Review. Vet. J..

[11] Mellor D.J. (2016). Moving beyond the “Five Freedoms” by Updating the “Five Provisions” and Introducing Aligned “Animal Welfare Aims. ” Animals.

[12] VanDierendonck M.C., Spruijt B.M. (2012). Coping in Groups of Domestic Horses—Review from a Social and Neurobiological Perspective. Appl. Anim. Behav. Sci..

[13] Hartmann E., Søndergaard E., Keeling L.J. (2012). Keeping Horses in Groups: A Review. Appl. Anim. Behav. Sci..

[14] Lesimple C., Fureix C., LeScolan N., Richard-Yris M.-A., Hausberger M. (2011). Housing Conditions and Breed Are Associated with Emotionality and Cognitive Abilities in Riding School Horses. Appl. Anim. Behav. Sci..

[15] Yarnell K., Hall C., Royle C., Walker S.L. (2015). Domesticated Horses Differ in Their Behavioural and Physiological Responses to Isolated and Group Housing. Physiol. Behav..

[16] Visser E.K., Ellis A.D., Van Reenen C.G. (2008). The Effect of Two Different Housing Conditions on the Welfare of Young Horses Stabled for the First Time. Appl. Anim. Behav. Sci..

[17] Ruet A., Lemarchand J., Parias C., Mach N., Moisan M.-P., Foury A., Briant C., Lansade L. (2019). Housing Horses in Individual Boxes Is a Challenge with Regard to Welfare. Animals.

[18] Siegel J., Augsburger C., Zollinger A., Bachmann I. (2018). Wie ist es, ein Pferd zu sein in der Schweiz?. Proceedings of the 13. Jahrestagung Netzwerk Pferdeforschung Schweiz.

[19] Hartmann E., Bøe K.E., Christensen J.W., Hyyppä S., Jansson H., Jørgensen G.H.M., Ladewig J., Mejdell C.M., Norling Y., Rundgren M. (2015). A Nordic Survey of Management Practices and Owners’ Attitudes towards Keeping Horses in Groups. J. Anim. Sci..

[20] Christensen J.W., Ladewig J., Søndergaard E., Malmkvist J. (2002). Effects of Individual versus Group Stabling on Social Behaviour in Domestic Stallions. Appl. Anim. Behav. Sci..

[21] Briefer Freymond S., Briefer E.F., von Niederhäusern R., Bachmann I. (2013). Pattern of Social Interactions after Group Integration: A Possibility to Keep Stallions in Group. PLoS ONE.

[22] Zilow V.K. (2015). Untersuchungen zur Haltung von Hengsten (Equus ferus caballus) in Bayern. Ph.D. Thesis.

[23] Steiner N. (2017). Untersuchung Zur Hengsthaltung in Niedersachsen. Ph.D. Thesis.

[24] Gehlen H., Krumbach K., Thöne-Reineke C. (2021). Keeping Stallions in Groups—Species-Appropriate or Relevant to Animal Welfare?. Animals.

[25] de Oliveira R.A., Aurich C. (2021). Aspects of Breeding Stallion Management with Specific Focus on Animal Welfare. J. Equine Vet. Sci..

[26] McDonnell S.M. (2008). Practical Review of Self-Mutilation in Horses. Anim. Reprod. Sci..

[27] Flauger B., Krueger K. (2013). Aggression Level and Enclosure Size in Horses (Equus Caballus). Pferdeheilkunde.

[28] Gmel A.I., Zollinger A., Wyss C., Bachmann I., Briefer Freymond S. (2022). Social Box: Influence of a New Housing System on the Social Interactions of Stallions When Driven in Pairs. Animals.

[29] Costa H., Fragoso S., Heitor F. (2019). The Relevance of Affiliative Relationships in Horses: Review and Future Directions. Pet Behav. Sci..

[30] Krueger K., Flauger B., Farmer K., Hemelrijk C. (2014). Movement Initiation in Groups of Feral Horses. Behav. Processes.

[31] Wolter R., Stefanski V., Krueger K. (2018). Parameters for the Analysis of Social Bonds in Horses. Animals.

[32] McDonnell S.M., Poulin A. (2002). Equid Play Ethogram. Appl. Anim. Behav. Sci..

[33] Christensen J.W., Søndergaard E., Thodberg K., Halekoh U. (2011). Effects of Repeated Regrouping on Horse Behaviour and Injuries. Appl. Anim. Behav. Sci..

[34] Grogan E.H., McDonnell S.M. (2005). Injuries and Blemishes in a Semi-Feral Herd of Ponies. J. Equine Vet. Sci..

[35] Jørgensen G.H.M., Borsheim L., Mejdell C.M., Søndergaard E., Bøe K.E. (2009). Grouping Horses According to Gender—Effects on Aggression, Spacing and Injuries. Appl. Anim. Behav. Sci..

[36] Mejdell C.M., Jørgensen G.H., Rehn T., Fremstad K., Keeling L., Bøe K.E. (2010). Reliability of an Injury Scoring System for Horses. Acta Vet..

[37] Keeling L.J., Bøe K.E., Christensen J.W., Hyyppä S., Jansson H., Jørgensen G.H.M., Ladewig J., Mejdell C.M., Särkijärvi S., Søndergaard E. (2016). Injury Incidence, Reactivity and Ease of Handling of Horses Kept in Groups: A Matched Case Control Study in Four Nordic Countries. Appl. Anim. Behav. Sci..

[38] Newberry R.C. (1995). Environmental Enrichment: Increasing the Biological Relevance of Captive Environments. Appl. Anim. Behav. Sci..

[39] Mason G.J. (1991). Stereotypies: A Critical Review. Anim. Behav..

[40] Fagen R. (1981). Animal Play Behavior.

[41] Hausberger M., Fureix C., Bourjade M., Wessel-Robert S., Richard-Yris M.-A. (2012). On the Significance of Adult Play: What Does Social Play Tell Us about Adult Horse Welfare?. Naturwissenschaften.

[42] Blois-Heulin C., Rochais C., Camus S., Fureix C., Lemasson A., Lunel C., Bezard E., Hausberger M. (2015). Animal Welfare: Could Adult Play Be a False Friend?. ABC.

[43] Heitor F., Vicente L. (2010). Dominance Relationships and Patterns of Aggression in a Bachelor Group of Sorraia Horses (Equus Caballus). J. Ethol..

[44] Waring G. (2002). Horse Behavior.

[45] Berger J. (1986). Wild Horses of the Great Basin: Social Competition and Population Size.

[46] Poncet P.-A., Bachmann I., Burkhardt R., Ehrbar B., Herrmann R., Friedli K., Leuenberger H., Lueth A., Montavon S., Pfammatter M. (2022). Ethical Reflections on the Dignity and Welfare of Horses and Other Equids—Pathways to Enhanced Protection. Summary Report.

[47] Mellor D.J. (2015). Positive Animal Welfare States and Reference Standards for Welfare Assessment. N. Z. Vet. J..

[48] Górecka-Bruzda A., Jaworski Z., Jaworska J., Siemieniuch M. (2020). Welfare of Free-Roaming Horses: 70 Years of Experience with Konik Polski Breeding in Poland. Animals.

[49] Raabymagle P., Ladewig J. (2006). Lying Behavior in Horses in Relation to Box Size. J. Equine Vet. Sci..

[50] Chung E.L.T., Khairuddin N.H., Azizan T.R.P.T., Adamu L. (2018). Sleeping Patterns of Horses in Selected Local Horse Stables in Malaysia. J. Vet. Beh..

[51] McGreevy P. (2004). Equine Behavior: A Guide for Veterinarians and Equine Scientists.

[52] Valenchon M., Petit O. Prise En Compte Du Bien-Être Social Pour Les Espèces Sociales: Pré-Étude Sur La Resocialisation d’un Groupe d’étalons. Proceedings of the Comprendre son Cheval, IFCE: Ecole Nationale d’Equitation.

[53] Löckener S., Reese S., Erhard M., Wöhr A.-C. (2016). Pasturing in Herds after Housing in Horseboxes Induces a Positive Cognitive Bias in Horses. J. Vet. Behav..

[54] Henry S., Fureix C., Rowberry R., Bateson M., Hausberger M. (2017). Do Horses with Poor Welfare Show ‘Pessimistic’ Cognitive Biases?. Sci. Nat..

[55] Lesimple C. (2020). Indicators of Horse Welfare: State-of-the-Art. Animals.

